# Transcriptomic profiling of *Trypanosoma congolense* mouthpart parasites from naturally infected flies

**DOI:** 10.1186/s13071-022-05258-y

**Published:** 2022-05-02

**Authors:** Sara Silva Pereira, Kawira Mathenge, Daniel Masiga, Andrew Jackson

**Affiliations:** 1 Department of Infection Biology and Microbiomes, Institute of Infection, Veterinary and Ecological Sciences, University of Liverpool, 146 Brownlow Hill, Liverpool, L3 5RF UK; 2 International Centre of Insect Physiology and Ecology, Nairobi, Kenya; 3 Faculdade de Medicina, Instituto de Medicina Molecular - João Lobo Antunes, Universidade de Lisboa, Lisbon, Portugal

## Abstract

**Background:**

Animal African trypanosomiasis, or nagana, is a veterinary disease caused by African trypanosomes transmitted by tsetse flies. In Africa, *Trypanosoma congolense* is one of the most pathogenic and prevalent causes of nagana in livestock, resulting in high animal morbidity and mortality and extensive production losses. In the tsetse fly, parasites colonise the midgut and eventually reach the mouthparts, from where they can be transmitted as the fly feeds on vertebrate hosts such as cattle. Despite the extreme importance of mouthpart-form parasites for disease transmission, very few global expression profile studies have been conducted in these parasite forms.

**Methods:**

Here, we collected tsetse flies from the Shimba Hills National Reserve, a wildlife area in southeast Kenya, diagnosed *T. congolense* infections, and sequenced the transcriptomes of the *T. congolense* parasites colonising the mouthparts of the flies.

**Results:**

We found little correlation between mouthpart parasites from natural and experimental fly infections. Furthermore, we performed differential gene expression analysis between mouthpart and bloodstream parasite forms and identified several surface-expressed genes and 152 novel hypothetical proteins differentially expressed in mouthpart parasites. Finally, we profiled variant antigen expression and observed that a variant surface glycoprotein (*VSG*) transcript belonging to *T. congolense* phylotype 8 (i.e. TcIL3000.A.H_000381200), previously observed to be enriched in metacyclic transcriptomes, was present in all wild-caught mouthpart samples as well as bloodstream-form parasites, suggestive of constitutive expression.

**Conclusion:**

Our study provides transcriptomes of trypanosome parasites from naturally infected tsetse flies and suggests that a phylotype 8 *VSG* gene is constitutively expressed in metacyclic- and bloodstream-form parasites at the population level.

**Graphical Abstract:**

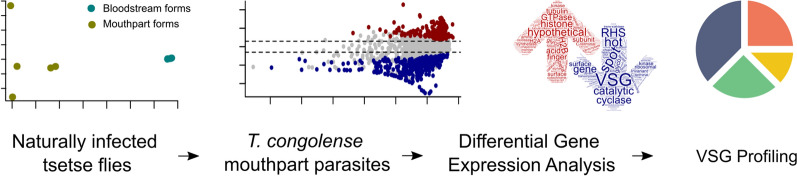

**Supplementary Information:**

The online version contains supplementary material available at 10.1186/s13071-022-05258-y.

## Background

Animal African trypanosomiasis (AAT), or nagana, is a livestock disease caused by the blood parasites African trypanosomes, particularly *Trypanosoma congolense*, *T. brucei*, and *T. vivax*, and transmitted by tsetse flies. In Africa, *T. congolense*, particularly of the savannah subtype, is one of the most pathogenic trypanosome species [1]. The life cycle of *T. congolense* alternates between the bloodstream of a mammalian host (usually livestock) and the tsetse fly vector. In the tsetse fly, the parasites first colonise the midgut as procyclic forms, but quickly cross the proventriculus towards the mouthparts of the fly. Here, they differentiate into epimastigotes and then into metacyclic forms, which are infective to mammals. Infection results in substantial animal mortality and economic loss. Animal productivity can be reduced by 38%, and there is a 12 and 10% loss in milk yield and animal returns, respectively [2]. Symptoms including anaemia and abortion lead to further production losses. With drug resistance widespread and vector control impractical, there is an urgent need for an AAT vaccine. However, a vaccine is considered impossible because of antigenic variation, a mechanism through which trypanosomes change their variant surface glycoproteins (VSG) to prevent a long-lasting, effective immune response. Previous vaccine attempts were directed at the bloodstream stage of the parasite. Yet, metacyclic parasites elicit the first immune response in the host. These parasites also express VSG on their surface, but they are not antigenically variant during an individual infection. A small set of VSGs has been found to be expressed by *T. congolense* metacyclic forms in three independent studies [3–5], which offers some possibility that this form of *T. congolense* may be amenable to vaccination.

Recently, we have developed variant antigen profiling, a method to describe and quantify the *VSG* repertoire for any isolate from sequencing reads [6]. Thus, we can examine metacyclic *VSG* expression to identify whether our *VSG* genes of interest are constitutively expressed in natural tsetse fly populations of distinct strains and genetic backgrounds. We collected wild tsetse flies from the Shimba Hills National Reserve (Kenya), diagnosed *T. congolense* mouthpart infections and sequenced their transcriptomes with the aim to analyse their expressed *VSG *repertoires. We found that gene expression profiles of trypanosomes from natural and experimental fly infections are distinct and we detected several genes differentially expressed between metacyclic- and bloodstream-form parasites. Importantly, we show that a particular *VSG* gene belonging to *T. congolense* phylotype 8 (i.e. TcIL3000.A.H_000381200) is expressed in both metacyclic- and bloodstream-form parasites at the population level. Further dissecting the dynamics of metacyclic *VSG* expression in natural trypanosome infections can help to identify constitutively expressed or metacyclic-specific *VSG* genes that may provide an effective vaccine target.

## Methods

### Sample collection, preparation, and sequencing

A total of 3237 flies were collected alive from the Shimba Hills National Reserve and its periphery using Vavoua traps [7] over the course of 10 days. Flies were killed by decapitation and midguts dissected to identify positive trypanosome infections. Mouthparts of flies with positive midguts were further dissected and diagnosed according to the description by Peel [8]. When metacyclic parasites were visible, the hypopharynx, labrum, and the parasite suspension that was released during dissection were collected and immediately frozen in liquid nitrogen. Sixty positive probosces were pooled into six groups of 10 flies. Total RNA was extracted using the AllPrep RNA/protein kit (Qiagen, UK) according to the manufacturer’s protocol, yielding RNA outputs of 108–374 ng per sample. Total RNA was used to prepare complementary DNA (cDNA) libraries for multiplexed spliced-leader sequencing using the *T. congolense* spliced leader sequence [9] as complementary strand amplification primer. The protocol described by Cuypers et al. (2017) [10] was followed up to library indexing. After amplification of the adaptor-ligated cDNA, we used the NEBNext^®^ Ultra™ II FS DNA Library Prep Kit (NEB) for fragmentation, indexing, and amplification, as previously described [11]. Samples were sequenced on the Illumina HiSeq 4000 platform as 2 × 150 base pair (bp) reads, yielding 1.5 to 2.6 × 10^7^ mappable reads per sample.

### Read processing and species identification

Paired reads were mapped to the tsetse fly [12], *T. brucei* Lister 427 [13], *T. congolense* IL3000 [14], and *T. vivax* Y486 [15] genomes using HISAT2 [16] under default settings. One sample yielded very few trypanosome-mappable reads and thus was not used in downstream analyses.

### Differential expression analysis

Reads mapping to *T. congolense* IL3000 were used in subsequent differential expression analyses. Alignment files were parsed through SAMtools (v1.9) [17] and transcript assembly and read count estimation were done using StringTie [18], following the developers’ protocol [19]. Transcripts with less than 10 read counts were removed using edgeR [20] and differential expression was estimated with limma-voom [21] and DESeq2 [22]. Transcripts were considered differentially expressed between parasite probosces and in the bloodstream if they had log2 fold change (logFC) > 1.5 and adjusted *P*-value < 0.01 in both limma-voom and DESeq2.

### Functional annotation of differentially expressed genes

Gene Ontology (GO) categories related to biological processes, molecular function, and cell components were identified using the annotated GO term list for the IL3000 genome available in TriTrypDB and topGO [23]. Enriched GO terms among differentially expressed (DE) gene products were established by Fisher’s exact test at a false discovery rate (FDR) *P*-value ≤ 0.05.

### Variant antigen profiling

For variant antigen profiling estimation, reads mapping to the tsetse genome were discarded. The remaining reads were assembled de novo using Trinity [24]. Transcript abundances were estimated in a reference-free manner using kallisto [25] as per the pre-compiled script available in the Trinity package. Assembled transcriptomes were screened for *VSG* transcripts using VAPPER [6], an automated tool for the analysis of *VSG* repertoires from genomic or transcriptomic sequencing data. It relies on a set of conserved protein motifs that allow the identification and quantification of each of the 15 phylotypes (i.e. phylogenetically related lineages) that comprise the *T. congolense* *VSG* repertoire [5].

## Results

### Tsetse flies circulating in the Shimba Hills National Reserve are mostly infected with *T. congolense*

Over the course of 10 days, we collected 3237 live tsetse flies, of which 108 (3.33%) had positive midgut infections. Of those, 62 (57.41%) had detectable metacyclic parasites in their mouthparts. By microscopy, and based on their larger size, we did not detect *T. vivax* parasites in the proboscis, only *T. congolense*. However, given the challenge in distinguishing African trypanosome species by microscopy, the possibility of mixed infections cannot be completely excluded. To address this, we performed whole-genome mapping.

We prepared six pools of 10 probosces, extracted RNA and sequenced them on the Illumina MiSeq platform. One of the samples was discarded post-sequencing due to low quality. For each sample, we obtained 7 to 13 million read pairs (mean ± standard error of the mean [SEM], 1.02E7 ± 9.73E05) (Table 1, of which 22.46% ± 3.99 mapped to the IL3000 genome and only 23.48% ± 2.70 mapped to the tsetse genome. As the trypanosome enrichment method performed at the cDNA library preparation stage (spliced-leader sequencing) cannot accurately select between African trypanosome species, it was necessary to check for contamination from *T. vivax* and *T. brucei*. We found a negligible number of reads mapping to these species (1.67% ± 0.23 and 0.46% ± 0.13, respectively), consistent with background noise. Therefore, we conclude that the probosces of the flies collected were mostly, if not only, colonised by *T. congolense*, and not by *T. vivax* or *T. brucei*.

To further investigate which *T. congolense* subspecies were present in our samples, we extracted all *T. congolense* reads mapping to *gapdh* gene sequences available from TriTrypDB or NCBI (TcIL3000.A.H_000478300 (savannah), TcIL3000.A.H_000155000 (savannah), TcIL3000.A.H_000478100 (savannah), TcIL3000.A.H_000775800 (savannah), AJ620287.1 (kilifi), AJ620288.1 (kilifi), AJ620289.1 (forest), AJ620286.1 (forest), AJ620285.1 (forest)). We found evidence of all three subspecies in our samples.

### Expression profiles of mouthpart parasites display wide variation

**Fig. 1:**
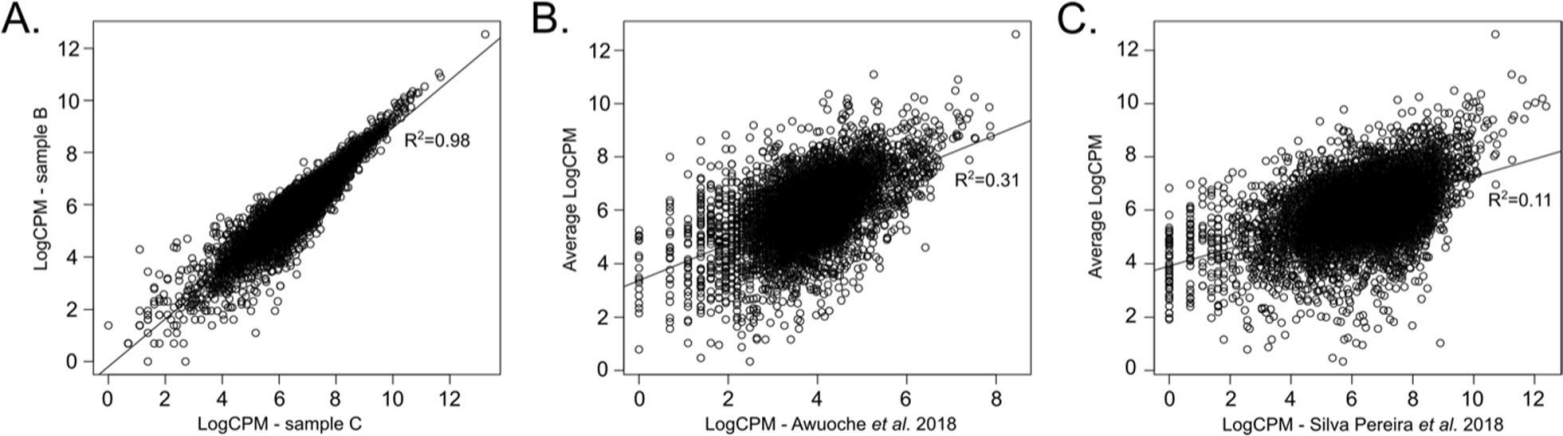
Correlation between transcriptomes of mouthpart trypanosomes from natural and experimental fly infections. **a** Correlation of transcriptomes from samples B and C (natural infections), shown as representatives of remaining samples (*r*^2^ = 0.98, Pearson’s correlation). **b** Correlation of average transcript abundance from transcriptomes obtained from natural infections and those presented by Awuoche et al. [27]. (*r*^2^ = 0.31, Pearson’’ correlation). **c** Correlation of average transcript abundance from transcriptomes obtained from natural infections and those presented by Silva Pereira et al. [5] (*r*^2^ = 0.11, Pearson’s correlation). *CPM* counts per million reads mapped

We selected reads mapped to the *T. congolense* IL3000 genome, assembled the transcriptomes of each pool, and compared them. We observed that they were reproducible and correlated well (average *r*^2^ = 0.94 ± 0.04, Pearson correlation). This shows that mouthpart parasites from naturally infected tsetse flies collected in the Shimba Hills National Reserve have similar transcriptomes. We then examined how the transcriptomes from parasites collected from wild flies compared to mouthpart parasites from experimental infections and found that such reproducibility is lost. Specifically, our data correlate weakly with the transcriptomes previously published by us [5] and by Awuoche et al. [26] (*r*^2^ = 0.11 and *r*^2^ = 0.31) (Fig. 1a–c). Nonetheless, variation is large even between experimental infections, as the correlation between these two previously published transcriptomes is also low (*r*^2^ = 0.10), despite similar experimental approaches. Correlation analysis of the most-abundant 1000 transcripts from the average read counts of natural infection transcriptomes does not improve correlation with those obtained from experimental infections (*r*^2^ = 0.09). This suggests that there is a wide variation in the expressed gene profile of mouthpart transcriptomes.

To further understand the differences between transcriptomes, we searched for differentially expressed genes between parasites collected from experimental and natural infections. We did not detect any differentially expressed genes between natural infection transcriptomes and those from Awuoche et al*.* [27]. This was surprising given the low correlation between the transcriptomes, and is likely a reflection of the low number of replicates in the latter dataset, which reduces the statistical power of the analysis. In contrast, we identified 757 differentially expressed genes (9.5%, corresponding to 321 upregulated and 436 downregulated genes) between the present natural infections and our previous experimental infections [5] (Additional file 1: Table S1). These differences may be explained by strain diversity, infection mode, tsetse rearing conditions, and number of pooled probosces, among other factors.

To better understand the variation in gene expression profiles between the natural and experimental datasets, we compared the experimental and natural infection transcriptomes to previously published bloodstream-form transcriptomes, using limma-voom [21]. We detected 316 differentially expressed (DE) genes in the mouthpart transcriptome produced by Awuoche et al*.* [27], 2672 in the sample previously published by us [5], and 1208 genes in the transcriptomes from the natural infections presented in the current study. However, only 110 of the DE genes are shared between all three datasets; 431 are shared between our experimental and natural infection datasets; 39 between Awuoche’s and our natural infection transcriptomes; and 104 are common to both experimental datasets.

Together, the strong correlation between the five samples collected from wild flies, the weak correlation between mouthpart transcriptomes of different sources, and the small overlap between the genes DE in the mouthparts compared to the bloodstream suggest that there is wide variation in the expressed gene profile of mouthpart transcriptomes. However, the weak correlation between the experimental datasets, indeed of the same magnitude as the correlation between natural and experimental datasets, suggests that such variation is not due to the wildness of the flies.

### Differential gene expression shows the abundance of hypothetical proteins upregulated in mouthpart parasites

**Fig. 2:**
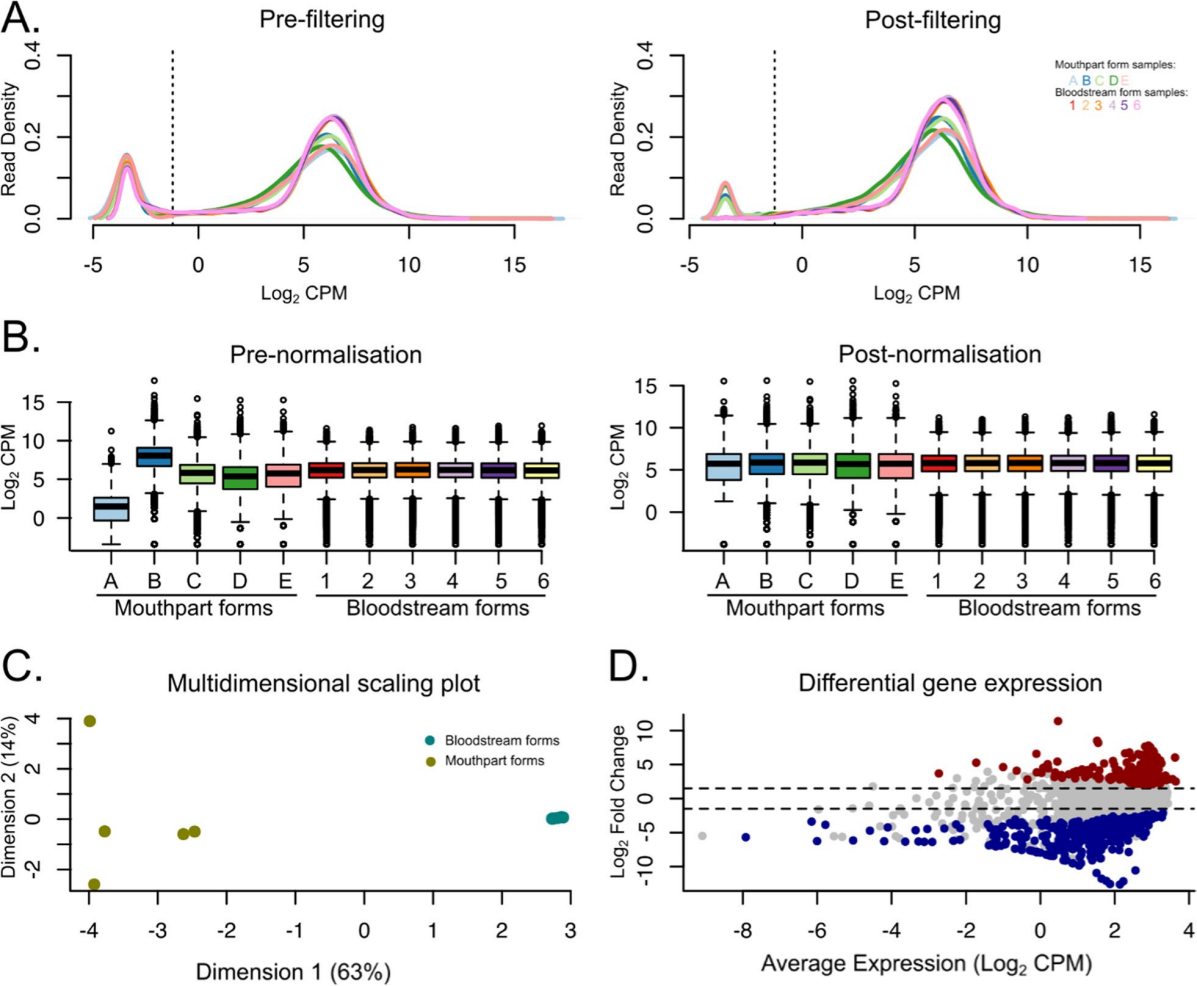
Differential gene expression analysis between mouthpart (MP) and bloodstream form (BSF) *T. congolense* parasites. **a** Read count density expressed as log-counts per million reads mapped (log_2_CPM) per sample before and after filtering transcripts with less than 10 read counts. **b** Distribution of log_2_CPM values per sample before and after normalisation for library size. **c** Multidimensional scaling plot showing the distances between gene expression profiles of each experimental group. **d** Volcano plot showing change in expression between conditions and transcript abundance, expressed as log_2_ fold change and log_2_cpm. Differentially expressed genes (log2FC >|1.5| and *P*-value < 0.01) are highlighted in blue (downregulated) or red (upregulated in mouthpart parasites)

Having established that trypanosomes from natural fly infections have a characteristic, but reproducible, expression profile, we proceeded to investigate how they differ from cultured *T. congolense* IL3000 bloodstream forms [28]. As previously described, these bloodstream-form transcriptomes were produced from parasites collected from the blood of infected laboratory mice at the first peak of parasitaemia and the ascending parasitaemia phase preceding it [28]. The library size (a proxy for sequencing depth) of mouthpart transcriptomes ranged between 2.48 × 10^6^ and 1.16 × 10^7^ per sample (average of 6.61 × 10^6^ ± 2.48 × 10^6^), whereas for bloodstream-form transcriptomes the library size ranged from 2.99 × 10^7^ to 3.57 × 10^7^ (average of 3.35 × 10^7^ ± 1.90 × 10^6^). Of the 10,315 transcripts annotated in the IL3000 genome, we detected 8835 transcripts with more than 10 read counts. Only these were considered in downstream analyses, as a measure to reduce the noise associated with low read counts (Fig. 2a). We have also normalised read counts for the differences in sequencing depth (Fig. 2b). As expected, we observed a clear separation between the expression profiles of mouthpart- and bloodstream-form parasites. The transcriptomes of mouthpart parasites are more variable than the bloodstream-forms, reflected by longer distances in the multidimensional scaling plot (Fig. 2c). This was predictable, as they potentially represent not only a mixture of parasite strains circulating in the Shimba Hills National Reserve, but also a mixture of parasites in different life stages (i.e. epimastigotes, pre-metacyclics, metacyclics).

**Fig. 3:**
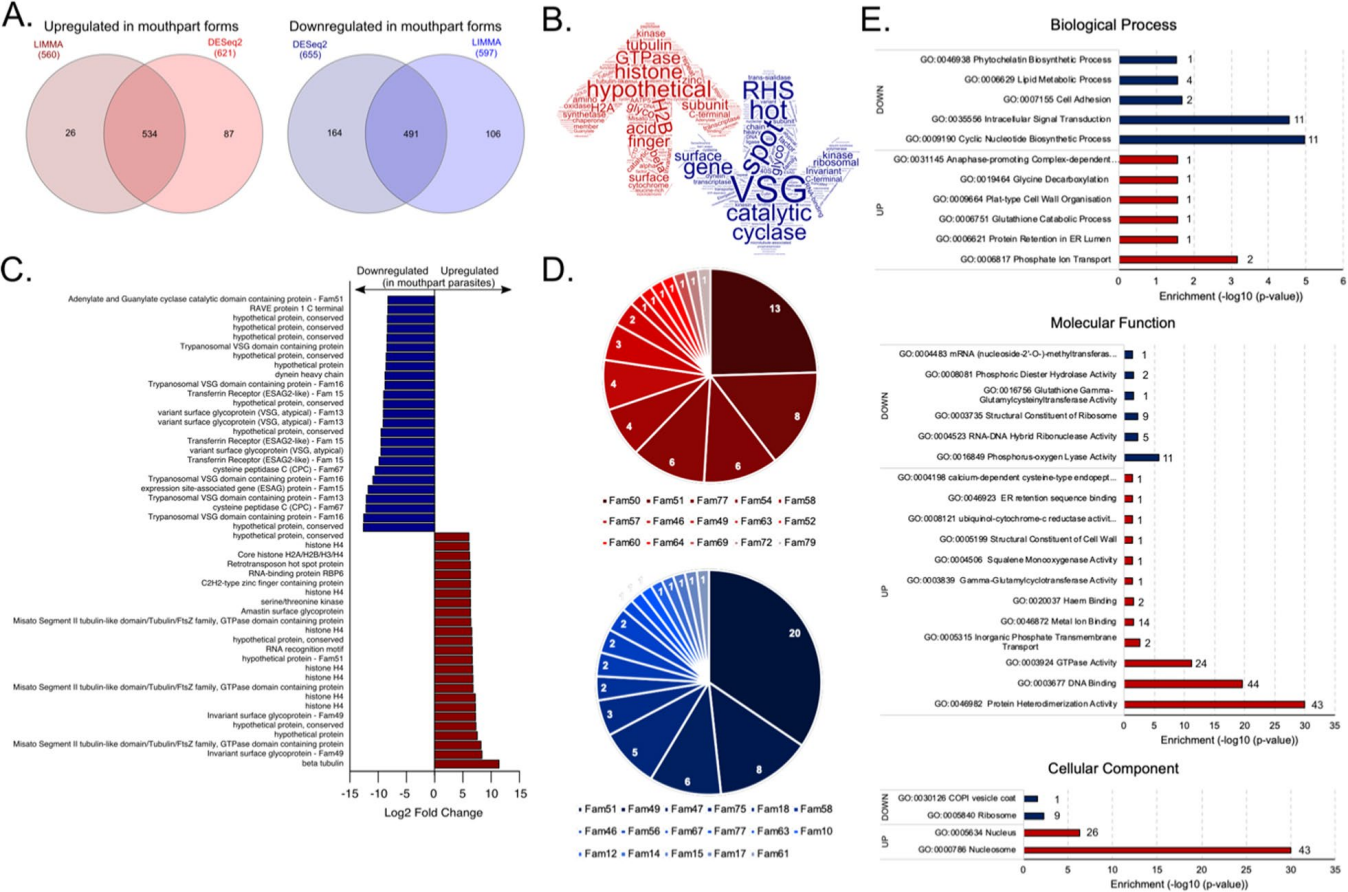
Differential gene expression analysis between mouthpart and bloodstream parasite forms. **a** Overlap between differentially expressed (DE) genes detected by limma and DESeq2. **b** Diagram showing the most represented words amongst the functional annotation of the differentially expressed genes. The size of the words is proportional to their frequency. **c** Annotation and fold change of the 25 genes most up- and downregulated in mouthpart parasites. **d** Frequency of DE genes belonging to non-*VSG* cell-surface gene families. Numbering in pie slices represent number of DE genes per family. Gene families are colour-coded according to key. Red represents genes upregulated in mouthpart parasites, whilst blue represents downregulated genes. **e** Gene Ontology (GO) enrichment analysis of differentially expressed genes between mouthpart and bloodstream parasite forms. Red represents upregulated GO terms in mouthpart parasites, blue represents downregulated. Numbers adjacent to the bars represent the number of significant genes that contributed to each GO term

We have subjected our samples to differential expression analysis using limma-voom [21] (Fig. 2d) and DESeq2 [22] (log2FC >|1.5| and *P*-value < 0.01). Limma-voom detected a total of 1208 differentially expressed genes (648 downregulated and 560 upregulated), whereas DEseq2 detected 1276 (Additional file 2: Table S2). Of these, 1062 were identified by both methods, corresponding to 88% and 83% of the total, respectively (Fig. 3a).

Our results suggest a strong contribution of epimastigote parasites to the mouthpart parasite population. The list of genes upregulated in mouthpart parasites is rich in hypothetical proteins, GTPase-related genes, zinc-finger proteins, and histones (Fig. 3b, red), which is consistent with previous observations in experimental tsetse fly infections [26]. Downregulated genes relate to *VSG*, several other surface genes, including ESAG2-like (most likely transferrin receptors), adenylate cyclases, ISG and RHS genes, and trans-sialidases (Fig. 3b, blue). This is also reflected within the top 25 most downregulated genes (Fig. 3c). Indeed, within the pool of DE genes, we found 111 genes to belong to 32 of the 74 previously described non-*VSG* cell-surface gene families [29]. Fam50, encoding brucei alanine-rich proteins (BARP), is the most represented cell-surface gene family amongst upregulated genes (*N* = 13), whereas Fam51, encoding adenylate cyclases, is the most common amongst downregulated genes (*N* = 20) Fig. 3d). Six gene families had members within both the up- and downregulated gene lists, which may indicate developmental regulation within these gene families. These were Fam46 (major surface protease–gp63), Fam49 (invariant surface glycoproteins), Fam51 (adenylate cyclases), Fam58 (MFS transporters), Fam63 (lipases), and Fam77 (hypothetical proteins conserved in *T. brucei*, *T. congolense*, and *T. vivax*).

We subjected the list of up- and downregulated genes to GO term enrichment analysis using topGO and the GO term annotation of the IL3000 genome available at TriTrypDB. We observed that the biological processes significantly enriched amongst the upregulated gene list are quite varied, including terms relating to mitosis, cell-wall organisation, glutathione catabolic process, or protein retention (Fig. 3e). In contrast, the downregulated processes relate to biosynthetic, metabolic, and signalling processes. In terms of molecular functions, we observed down-regulation of ribosome processes and mostly upregulation of DNA binding, protein heterodimerization activity and enzymatic functions (Fig. 3e). The enrichment analysis of cellular components also reflects lower protein synthesis in mouthpart forms, which is consistent with the quiescent state of metacyclic parasites (Fig. 3e). In contrast, there seems to be an increase in nucleus and nucleosome activity in mouthpart parasites, which was also observed by Awuoche et al. [26] in their comparison with cardia parasites. The GO term analysis confirms that mouthpart forms are less physiologically active than bloodstream parasite forms.

Interestingly, we found that 143 out of the 511 upregulated genes and 144 of the 491 downregulated genes are annotated as hypothetical proteins. Whilst 135 of these hypothetical proteins have already been detected by Awuoche et al. [26] as differentially expressed in mouthpart parasites compared to cardia parasites, we found 83 additional hypothetical proteins that are downregulated and 69 that are upregulated in mouthpart parasites compared to bloodstream forms (Additional file 3: Table S3).

### At the infrapopulation level, variant antigen profiles of *T. congolense* metacyclic parasites show a partly reproducible expression pattern

**Fig. 4:**
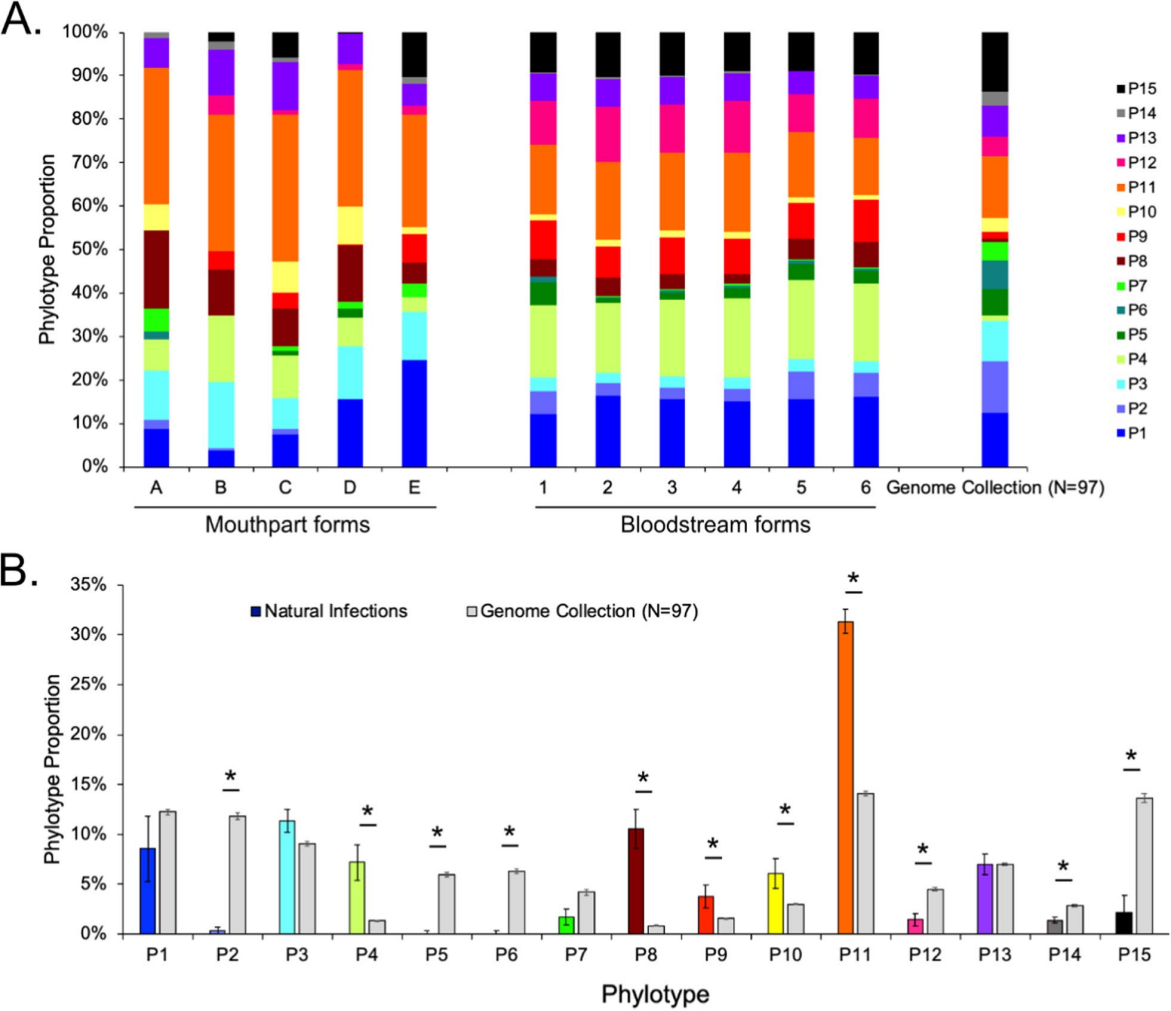
Variant antigen profiles of *T. congolense* are non-random and partially reproducible. **a** Variant antigen profiles of *T. congolense* metacyclic parasites collected from tsetse flies circulating in the Shimba Hills National Forest and of bloodstream-form parasites from culture [28]. **b** Comparison of average phylotype proportion (adjusted for transcript abundance) in transcriptomic samples from naturally infected, fly-derived *T. congolense* metacyclics and the average *VSG* genomic profile of *T. congolense* [6] (mean ± SEM). Statistical analysis reveals that phylotype (P)2, P5, P6, P12, P14, and P15 are underrepresented in the transcriptomes (independent *t*-test, *P*-value < 0.001), while P4, P8, P9, P10, and P11 are significantly overrepresented (independent *t*-test, *P*-value < 0.001) in fly-derived metacyclic transcriptomes. Stars indicate statistically-significant results (independent *t*-test)

We then proceeded to investigate the metacyclic *VSG* repertoire and how it differs from that of bloodstream forms. An infected proboscis contains epimastigotes, metacyclic parasites, and a small percentage of long dividing trypomastigotes migrating from the cardia [30]. Whilst distinguishing between epimastigote and metacyclic transcriptomes is not possible without parasite purification or enrichment, we can analyse metacyclic *VSG* expression because *VSG* genes are not expressed by epimastigotes. We estimated the variant antigen profiles of each sample with VAPPER [6] (Fig. 4a). VAPPER allows the comparison of *VSG* diversity by searching for diagnostic protein motifs that allow grouping of individual *VSG* genes into 15 conserved phylotypes. By comparing the representation of individual phylotypes, we can detect changes in *VSG* expression patterns [5]. In the present study, we prepared pools of 10 probosces to ensure that we obtained sufficient material for reliable transcriptomes. As such, we committed to profiling the *VSG* repertoire across a cohort, rather than a clonal population. We observed that all five samples have an overall similar pattern of *VSG* expression, which is consistent with previous observations from experimental infections [5]. However, we detected subtle fluctuations in *VSG* phylotype (P) expression patterns (Fig. 4a). Specifically, we observed the highest variation in the abundance of P1, P8, P4, P15, and P10, whereas P14, P6, P5, P2, and P12 were the least variable between samples. We also estimated *VSG* diversity in the bloodstream-form parasites. These displayed a very high degree of reproducibility between replicates, reflecting their origin from a single infection with the same blood stabilate [28].

When we compared the expressed *VSG* profiles of metacyclic parasites to the average genomic *VSG* profile of *T. congolense* (estimated from 97 genomes, available from the VAPPER database [6]), quite considerable variation was revealed. We observed that all phylotypes, with the exception of P1, P3, P7, and P13, were statistically different (independent *t*-test, *P*-value < 0.01) (Fig. 4b). P2, P5, P6, P12, P14, and P15 were less abundant in the transcriptomes than in their relative frequency in the genomic repertoire (independent *t*-test, *P*-value < 0.001), whilst P4, P8, P9, P10, and P11 were more abundant (independent *t*-test, *P*-value < 0.01). The differences in abundance of phylotypes P4, P7, P8, P9, P11, P12, and P15 are consistent with previous observations from experimental infections with two different strains (1/148 and TC13) [5, 6].

The contribution of phylotype 8 to the expressed *VSG* profiles of metacyclic parasites (10.85% ± 1.94) is noteworthy, because it represents a very small proportion of *VSG* genes in the genome (0.94% ± 0.09), and likewise to the expressed repertoires of bloodstream-form parasites (3.98% ± 0.46). Upon more detailed analysis, we discovered that its abundance is mostly due to the high expression of gene TcIL3000.A.H_000381200 (formerly TcIL3000_0_09520). This *VSG* transcript is present in all samples from both mouthpart- and bloodstream-form parasites. Despite being slightly more abundant in metacyclic than in bloodstream-form parasites, it is not differentially expressed (log_2_FC = 2.32, *P*-value = 0.12).

## Discussion

*T. congolense* is one of the most prevalent and pathogenic African trypanosome species in the ‘tsetse belt’ [1, 31,33]. Its development in the tsetse fly is essential for transmission and successful establishment in the mammalian host. In this work, we produced the first transcriptomes of mouthpart *T. congolense* parasites collected from naturally infected, wild tsetse flies, compared them to available transcriptomes of mouthpart- and bloodstream-form parasites, and described their variant antigen repertoires. We showed that transcriptomes from mouthpart infections display large variability. As a consequence, transcriptomes from experimental infections might not be a good proxy of wild trypanosomes, either because experimental conditions do not recapitulate any natural setting, or because natural conditions are sufficiently diverse that most do not recapitulate standard experimental conditions.

Our results also highlight the importance of surface-expressed genes in the adaptation of *T. congolense* to the different host or vector environments. In particular, we observed considerable developmental regulation amongst cell-surface phylome families (including Fam 46 (major surface protease–gp63), Fam49 (invariant surface glycoproteins), Fam51 (adenylate cyclases), Fam58 (MFS transporters), Fam63 (lipases), and Fam77 (hypothetical proteins conserved in *T. brucei*, *T. congolense* and *T. vivax*). Surface-expressed genes are responsible for many of the species-specific adaptations of trypanosomes. This was perceived in the initial comparative genomics analyses [15, 38], as gene families encoding cell-surface proteins are often non-homologous and lineage-specific [29]. Furthermore, the fact that we have identified 152 novel hypothetical proteins differentially expressed in mouthpart parasites shows that there is still much hidden gene functionality waiting to be described in trypanosomes.

We observed that the variant antigen profiles of both metacyclic- and bloodstream-form parasites were reproducible across biological replicates. This reproducibility is remarkable, given the potential diverse origin of the mouthpart parasites, but agrees with preceding findings from experimental infections [5]. These results, while relating to a mixture of strains rather than individual infections as previously, corroborate what we saw on that occasion. That is, a specific, rare VSG is observed in all mouthpart transcriptomes, and the metacyclic *VSG* repertoire of the parasite population is, therefore, at least partly predictable. The most parsimonious explanation for this observation is that *T. congolense* *VSG* genes are not randomly selected from the genome repertoire for expression in the metacyclic life stage. This concept of *VSG* expression obeying a predetermined, probabilistic order agrees with current knowledge of *T. brucei* [39, 40] and *T. vivax* [11] antigenic variation in the mammalian host, but is distinct from the current understanding of *T. brucei* metacyclic *VSG* expression. In *T. brucei*, metacyclic VSGs do not seem to follow a predetermined order of expression, which results in variable expression patterns, that are not reproducible over time in either natural or experimental conditions [41]. What remains unclear is whether expression patterns and the probabilistic order of VSG expression exist at the single-cell level. Single-cell RNA sequencing of *T. congolense* metacyclic parasites will be able to address this question.

We have identified one *VSG* gene, belonging to P8, that is expressed in both metacyclic- and bloodstream-form parasites (TcIL3000.A.H_000381200, formerly TcIL3000_0_09520). The same *VSG* transcript was detected in experimental metacyclic-form transcriptomes in two independent experiments [5, 26]. This suggests that P8 is not metacyclic-specific as we previously suggested, but perhaps constitutively expressed throughout the life cycle. Nevertheless, a constitutive expression profile for P8 is still incompatible with the expected expression profile of an antigenically variant *VSG*. We should not observe a *VSG* gene expressed in all flies at all times if it functions as a variant antigen because such gene, translated into an antigen, would undermine the variable immune stimulation upon which the immune evasion is based. This represents a definite difference with *T. brucei* metacyclic *VSG*, among which there are no sequence types that can be predicted to be always present [42].

In 2018, when we first discovered P8 to be highly represented in the variant antigen profiles of metacyclic parasites from experimental fly infections [5], we postulated that this gene(s) could be potentially exploitable for vaccine design, if reproducible in nature. Now, we confirm that TcIL3000.A.H_000381200 is enriched in natural fly infections and is also expressed by bloodstream-form parasites. If this *VSG* is expressed constitutively, it may have evolved a distinct function required by cells not involving antigenic variation. Examples of *VSG* genes acquiring novel functions within trypanosome genomes are plentiful [43]. They include ESAG2 and the transferrin receptors, both surface proteins expressed in the cell body and the flagellar pocket, respectively, during the bloodstream-form stage [44]. To consider TcIL3000.A.H_000381200 a promising vaccine candidate, it must encode an immunogenic surface protein. Despite the common belief that effective vaccines against African trypanosome infections are virtually impossible to design due to the very successful immune evasion strategies employed by the parasites [45], researchers have recently discovered an invariant surface antigen that induces long-lasting, protective immunity to *T. vivax* infections in mice [46]. In another study, single-cell RNA sequencing of *T. brucei* mouthpart forms revealed a metacyclic-specific surface-expressed gene that showed potential as a transmission-blocking antigen (i.e. SGM1.7, belonging to Fam10) [47]. Therefore, as we continue to dissect the variability of surface-expressed genes, the possibility of a vaccine against trypanosomiasis becomes more plausible.

In conclusion, this study provides insight into the gene expression profile of *T. congolense* mouthpart forms in an epidemiological and biologically relevant context. Our results expose the need to dissect the functionality of novel hypothetical proteins, numerous genes encoding putative cell-surface proteins, and an apparently constitutively expressed *VSG* gene, as they may be promising candidates for drug treatment and vaccinology strategies against trypanosomiasis.

**Table 1:** Sequencing and mapping statistics for transcriptomes produced from mouthpart parasites collected at the Shimba Hills National Reserve (Kenya)

Sample	Number of reads (#)	# Tsetse	% Tsetse	# IL3000	% IL3000	# TvY486	% TvY486	# Tb427	% Tb427
A	1.29E+07	2.22E+06	17.23%	3.22E+06	24.95%	2.71E+05	2.10%	5.23E+04	0.41%
B	9.72E+06	1.98E+06	20.34%	2.34E+06	24.07%	1.23E+05	1.27%	2.40E+04	0.25%
C	1.29E+07	2.36E+06	18.32%	4.78E+06	37.07%	1.89E+05	1.46%	1.32E+05	1.02%
D	1.07E+07	3.27E+06	30.43%	1.33E+06	12.38%	2.59E+05	2.41%	4.93E+04	0.46%
E	7.08E+06	2.20E+06	31.09%	9.79E+05	13.82%	7.76E+04	1.10%	1.22E+04	0.17%
Min	7.08E+06	1.98E+06	17.23%	9.79E+05	12.38%	7.76E+04	1.10%	1.22E+04	0.17%
Max	1.29E+07	3.27E+06	31.09%	4.78E+06	37.07%	2.71E+05	2.41%	1.32E+05	1.02%
Mean	1.07E+07	2.41E+06	23.48%	2.53E+06	22.46%	1.84E+05	1.67%	5.39E+04	0.46%
SEM	9.73E+05	2.01E+05	2.70%	6.14E+05	3.99%	3.36E+04	0.23%	1.87E+04	0.13%

Read mapping was performed with HISAT2 [16]

## Supplementary Information

**Additional file 1. **

**Additional file 2. **

**Additional file 3.**

## Data Availability

The datasets generated and/or analysed during the current study are available in NCBI/SRA, under project accession number PRJNA795045.
